# Oxidation of carcinogenic 2-nitroanisole by rat cytochromes P450 – similarity between human and rat enzymes

**DOI:** 10.2478/v10102-010-0035-x

**Published:** 2010-11

**Authors:** Martina Svobodová, Helena Dračínská, Markéta Martínková, Jiří Hudeček, Petr Hodek, Eva Frei, Marie Stiborová

**Affiliations:** 1 Department of Biochemistry, Faculty of Science, Charles University, Prague, Albertov 2030, 128 40 Prague 2, CZECH REPUBLIC; 2 Division of Molecular Toxicology, German Cancer Research Center, Im Neuenheimer Feld 280, 69120 Heidelberg, GERMANY

**Keywords:** 2-nitroanisole, 2-nitrophenol, metabolism, detoxication, oxidation, cytochrome P450

## Abstract

2-Nitroanisole (2-NA) is an important industrial pollutant and a potent carcinogen for rodents. Understanding which cytochrome P450 (CYP) enzymes are involved in its metabolism are important to assess an individual's susceptibility to this environmental carcinogen. The aim of this study was to evaluate the efficiency of rat hepatic CYPs to oxidize 2-NA, to examine the metabolites formed during such an oxidation, and to compare such efficiencies of rat CYPs with those of human. 2-NA is oxidized by rat hepatic microsomes to 2-nitrophenol (2-NP) as the major metabolite, and to 2,6-dihydroxynitrobenzene (2,6-DNB) and 2,5-dihydroxynitrobenzene (2,5-DNB) as the minor products. All these metabolites are suggested as detoxication products. Using hepatic microsomes of rats pre-treated with specific CYP inducers and microsomes from Baculovirus transfected insect cells expressing recombinant rat and human CYP enzymes we found that rat recombinant CYP2E1, 2D2, 2B2, 2C6 and 1A1, as well as orthologous human CYP enzymes are the most efficient enzymes metabolizing 2-NA. However, human CYP1A1 oxidize 2-NA with a higher efficiency than the enzyme of rats. The results show the participation of orthologous CYPs in 2-NA oxidation by both species and underline the suitability of rat species as a model to evaluate human susceptibility to 2-NA.

## Introduction

Aromatic nitro-compounds are potent toxic or carcinogenic compounds, presenting a considerable danger to the human population (Garner *et al*., 1984; IARC, 1989). They are widely distributed environmental pollutants found in workplaces (e.g. in chemical industry), in emissions from diesel and gasoline engines and on the surface of ambient air particulate matter (IARC, 1989), where they add to local and regional pollution (car exhausts, technological spills). The toxicity and carcinogenicity of these compounds, their metabolic pathways and the persistence of residues of these compounds and/or their metabolites in organisms have been examined (IARC, 1998, Purohit and Basu, 2000). However, the knowledge of the fate of several aromatic nitro compounds and their physiological effects in humans is still scarce (Purohit and Basu, 2000).

2-Nitroanisole (2-methoxynitrobenzene, 2-NA) is used primarily as a precursor in the synthesis of *o*-anisidine (2-methoxyaniline), an intermediate in the manufacture of many azo dyes (NTP 1978; 1993). 2-NA and *o*-anisidine exhibit strong carcinogenic activity, causing neoplastic transformation in the urinary bladder, and to a lesser extent, in the spleen, liver and kidneys in rodents (NTP 1978; 1993). 2-NA is also a toxic compound, causing anemia. The anemia is characterized by increased levels of methemoglobin and accelerated destruction of erythrocytes (NTP 1978). In 1993, an industrial accident in the Hoechst Company in Germany led to a large-scale leakage of 2-NA and subsequent local and regional contamination. Xanthine oxidase (XO) is the principal enzyme responsible for the reductive metabolism of 2-NA, catalyzing the formation of *N*-(2-methoxyphenyl)hydroxylamine and *o*-anisidine (Mikšanová *et al*., 2004a; Stiborová *et al*., 2004). Deoxyguanosine adducts derived from *N*-(2-methoxyphenyl)hydroxylamine were found *in vivo* in DNA of several tissues, mainly urinary bladder, of rats treated with 2-NA as well as *in vitro* after incubation of 2-NA and DNA with human hepatic cytosols or buttermilk XO (Stiborová *et al*., 1998; 2004). In contrast, 2-NA oxidation by microsomal CYP enzymes from human and rabbit to 2-NP and dihydroxyderivatives of this metabolite leads to its detoxication (Mikšanová *et al*., 2004b; Dračínská *et al*., 2006).

The present study was undertaken to investigate 2-NA oxidation by rat microsomal CYP enzymes in detail. Another aim of this study was to compare the capability of rat CYPs oxidizing 2-NA with those of humans.

## Materials and methods

### Enzymes

Supersomes™, microsomes isolated from insect cells transfected with baculovirus constructs containing cDNA of one of the following human and/or rat CYPs: CYP1A1, 1A2, 1B1, 2A2, 2A6, 2B2, 2B6, 2C6, 2C8, 2C9,2C11, 2C12, 2C13, 2C19, 2D1, 2D2, 2D6, 2E1, 3A1, 3A2, 3A4 with cytochrome b_5_ and expressing NADPH:CYP reductase were from Gentest Corp. (USA).

### Animal experiments and preparation of microsomes

The study was conducted in accordance with the Regulations for the Care and Use of Laboratory Animals (311/1997, Ministry of Agriculture, Czech Republic), which complies with Declaration of Helsinki. Microsomes from livers of ten male untreated Wistar rats and those of rats pretreated with β-NF (Sigma, UK) and PB were prepared by the procedure described previously (Stiborová *et al*., 2002; Dračínská *et al*., 2006). Rat liver microsomes contained 0.6nmol CYP/mg protein. Hepatic microsomes of rats treated with β-NF and PB contained 1.3 and 1.5nmol CYP/mg proteins, respectively.

### Incubations

Unless state otherwise, incubation mixtures used for studying 2-NA metabolism were as described previously (Mikšanová *et al*., 2004b; Dračínská *et al*., 2006). Incubation mixtures, in which the efficiencies of Supersomes expressing human and rat CYPs were tested, were the same except that only 10 pmol of CYP was used. Incubations that were used for investigating the time-dependence of 2-NA oxidation by rat hepatic microsomes contained 0.1mM 2-NA dissolved in methanol. In all other incubations 1 mM 2-NA was used. The 2-NA metabolites were separated by HPLC and detected by UV absorption as described (Stiborová *et al*., 2004; Dračínská *et al*., 2006).

## Results

2-NA is oxidized by rat hepatic microsomes to 2-nitrophenol (2-NP), 2,5-dihydroxynitrobenzene (2,5-DNB) and 2,6-dihydroxynitrobenzene (2,6-DNB), which were found to be detoxication 2-NA metabolites (Mikšanová *et al*., 2004b; Dračínská *et al*., 2006). 2-NP is the major metabolite generated by rat hepatic microsomes. Therefore, hepatic microsomal CYP enzymes participate in detoxication of this environmental carcinogen. Besides microsomes isolated from livers of uninduced rats, those from rats treated with inducers of individual CYPs, β-naphtoflavone (β-NF, which induces CYP1A1/2) and phenobarbital (PB, which induces CYP2B1/2) were used in the experiments. These experiments might increase our knowledge which CYP enzymes are involved in 2-NA oxidation in rat liver.

**Figure 1:**
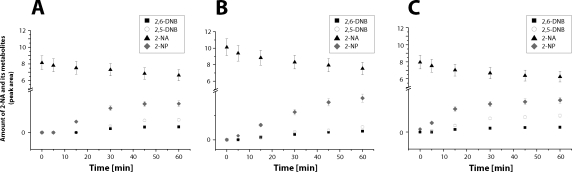
Time-dependence of 2-NA oxidation by rat hepatic microsomes of untreated rats (**A**), by those induced with β-NF (**B**) and PB (**C**). Incubation mixtures were of the same composition as those described in Materials and methods, but 0.1mM 2-NA was used.

**Figure 2:**
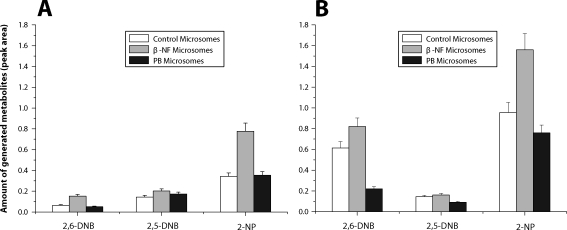
Metabolites formed by oxidation of 2-NA (0.1 mM, **A**) and (1 mM, **B**) by rat hepatic microsomes.

Oxidation of 2-NA to 2-NP, 2,5-DNB and 2,6-DNB was time-dependent. At the 0.1 mM concentration of 2-NA, the reaction (consumption of 2-NA and production of 2-NP, 2,5-DNB and 2,6-DNB) was linear up to 15–20 min (Figure 1), whereas when 2-NA was present in incubations at 1mM concentrations, the reaction was linear up to 45min (not shown). Incubations of 2-NA with liver microsomes of rats treated with β-NF led to an increase in 2-NA oxidation to 2-NP and 2,6-DNB, but almost no effect on production of 2,5-DNB was found in these microsomes. On the contrary, incubations of 2-NA with hepatic microsomes from rats pre-treated with PB led to a decrease in formation of 2-NA metabolites (Figure 2).

**Figure 3:**
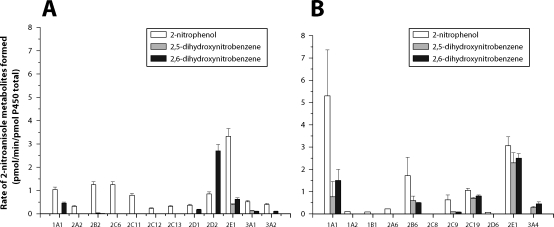
Metabolites formed by oxidation of 2-NA (1 mM) by recombinant CYPs of rat (**A**) and human (**B**).

To characterize further the role of individual CYPs in oxidation of 2-NA, rat recombinant CYP enzymes were used as the oxidation system. The data found with these rat oxidation enzymes were additionally compared with those found with human recombinant CYPs. Using microsomes of Baculovirus transfected insect cells containing recombinant rat or human CYPs and NADPH:CYP reductase (Supersomes™), we found that rat CYP2E1 followed by CYP2D2, 2C6, 2B2, and 1A1 were the most effective in 2-NA oxidation (Figure 3). Similarly, human CYP2E1, 2B6, 2C19 and 2C9 were also very effective to oxidize 2-NA, human recombinantCYP1A1 was, however, the most efficient human recombinant CYP oxidizing this carcinogen (Figure 3).

## Discussion

The results found in the present study show that rat hepatic microsomes efficiently oxidize industrial pollutant and potential human carcinogen (Stiborová *et al*., 2004), 2-NA, to the detoxication metabolites 2-NP, 2,5-DNB and 2,6-DNB. The same 2-NA metabolites we found in this work were also formed by human hepatic microsomes (Mikšanová *et al*., 2004b; Dračínská *et al*., 2006). The present study also documents the role of specific rat CYP enzymes in oxidative pathways of 2-NA. The most effective enzyme responsible for 2-NA metabolism was rat recombinant CYP2E1 followed by CYPs of 2D, 2C, 2B and 1A subfamilies. The human recombinant CYP enzymes of analogous subfamilies were also effective in 2-NA oxidation. Moreover, the CYP2E1 enzyme was also the most important CYP, oxidizing 2-NA in human livers (Mikšanová *et al*., 2004b; Dračínská *et al*., 2006). This finding indicates a similarity among CYPs metabolizing 2-NA in humans and rats. Therefore, we suggest that the results obtained with a rat animal model should provide some indication of what might occur with 2-NA in humans. 2-NA causes tumours in this animal model (NTP, 1993). The finding present in this and former studies (Stiborová *et al*., 1998; 2004; Mikšanová *et al*., 2004b; Dračínská *et al*., 2006) might be one of the criteria important to show that rats might be a suitable model to predict human metabolic susceptibility to 2-NA. This is important in view of the evaluation of 2-NA carcinogenicity as a carcinogenic risk factor for humans, particularly persons exposed to 2-NA during azo dye production. The analysis of 2-NA metabolites in urine of such individuals as well as the determination of 2-NA-derived DNA adducts in lymphocytes should be used to monitor these workers. Nevertheless, also other criteria, such as the type of tumour or organs of tumorigenesis, might be even more important to estimate 2-NA carcinogenicity to human. Therefore, the extensive examination of these persons for the organ specific cancer development should also be monitored.

However, in the case of human recombinant CYPs, CYP1A1 was much more effective to oxidize 2-NA that its rat orthologous enzyme. Nevertheless, the CYP1A inducer, β-NF, induced a pronounced increase in 2-NA oxidation by microsomes of rats treated with this compound, indicating importance of CYP1A for 2-NA oxidation in rat livers. Now, we can only speculate on the discrepancies found between the rat microsomal system and rat recombinant CYP1A1. The study investigating the effect of other compounds of the mixed-function oxidase system in microsomes, such as cytochrome b_5_, influencing the CYP1A1-mediated oxidation of some xenobiotics (Stiborová *et al*., 2006), can shed some light to explain this discrepancies.

In conclusion, the present study showing the similarities of CYP-mediated oxidation of 2-NA in rat and human livers is promising for further studies with a rat animal model to investigate the mechanism of 2-NA carcinogenicity, which might be important for risk assessment of this carcinogen for humans.
